# Supplementary material for: Effects of psychogenic stress on some peripheral and central inflammatory markers in rats with the different level of excitability of the nervous system
Source: PLoS One. 2021 Jul 29;16(7):e0255380. doi: 10.1371/journal.pone.0255380 (PMC8321229; doi:10.1371/journal.pone.0255380)
Supplement: S1 Table — (DOCX) [file pone.0255380.s001.docx]

**S_6. The direction of poststress changes in the studied parameters in rats of two strains**

| parameters | behaviour | | | N:L ratio | | | Microglia in HIP | | | | | | | | |
| --- | --- | --- | --- | --- | --- | --- | --- | --- | --- | --- | --- | --- | --- | --- | --- |
|  | 1 d | 7 d | 24 d | 1 d | 7 d | 24 d | CA1 | | | CA3 | | | DG | | |
|  |  |  |  |  |  |  | 1 d | 7 d | 24 d | 1 d | 7 d | 24 d | 1 d | 7 d | 24 d |
| HT strain | --- | --- | --- |  | --- | --- | --- |  | --- | --- | --- | --- | --- | --- | --- |
| LT strain | exploratory | locomotion | exploratory |  |  | --- | --- |  | --- | --- |  | --- | --- |  | --- |
