# Supplementary material for: Effects of psychogenic stress on some peripheral and central inflammatory markers in rats with the different level of excitability of the nervous system
Source: PLoS One. 2021 Jul 29;16(7):e0255380. doi: 10.1371/journal.pone.0255380 (PMC8321229; doi:10.1371/journal.pone.0255380)
Supplement: Attachment — Submitted filename: Response to Rev 2.docx [file pone.0255380.s007.docx]

Thank you for your careful reading of our work and valuable comments.

Below are our answers to each of them.

1. Please identify the member family of TNF in this statement, or the author is referring to the TNF as a superfamily "tumor necrosis factor (TNF) are significantly increased in the blood of patients with posttraumatic stress disorder (PTSD)."

Done

2. Animals and stress protocol. Please add the animal's age and the Wistar strain in the first line of the paragraph to facilitate the reader. The number of animals per group, the total number of animals, and if they had mortality.

Done

3. I suggest to the authors to add a timeline.

Done (Fig 1)

4. I suggest to the author add the details of the levels of CNS excitability of the animals. The value of that intensity above which the neuron spikes (top) and the voltage threshold.

We have added the necessary explanations to the methods section. Also in the Results section, we added a picture showing significant differences in the threshold volage levels in these two rat strains.

The study of evoked potentials in brain structures after electrical stimulation of the tibial nerve is a special task. But we have recently demonstrated contrasting differences between the strains in terms of the evoked neuronal activity of the amygdaloid complex (Prefrontal cortex electrical stimulation). Link to the article by Sivachenko, 2020 is given.

5. Why did the LT rat's anxiety-like symptoms in the EPM test disappeared?

From the data we obtained, it is impossible to conclude about the reason for such dynamics in behavior, but we made an assumption about the involvement of compensatory mechanisms (added to the discussion).

6. Animals react to stress by activating the hypothalamic-pituitary-adrenocortical axis (HPA) and releasing glucocorticosteroids from the adrenal cortex. What is the mechanism that stress increases the number of neutrophils?

Endogenous GCs are one of the factors that promote the maturation of neutrophils in the bone marrow and favor the mobilization of neutrophils from the bone marrow into circulation. Another mechanism is neutrophil resistance to GC-induced apoptosis, which prolongs their life span. We have added an explanation to the discussion text.

7. Did the author evaluate cytokines/chemokines and other inflammatory mediators in the animals.

Of course, it is necessary to evaluate other markers of neuroinflammation. We plan to evaluate the level of proinflammatory cytokines in the brain and blood of these animals in our next studies.

8. Why did the microglial cells increased the number by are in the resting morphology?

Based on our data, we cannot draw conclusions about the state of the microglia (activated\resting).

9. Why did the microglial cells increased in number but remained in the resting shape?

From our data, we cannot conclude which phenotype (activated or resting) has microglia in the hippocampus. In order to answer this question, it is necessary to study other markers of neuroinflammation (cytokines) and the morphology of microglial cells. We noted this in the discussion.

10. I suggest a flow chart to explain the conclusion.

The table “**The direction of poststress changes in the studied parameters in rats of two strains”** was added in Supplement (S6).
